# ALBERT-Based Self-Ensemble Model With Semisupervised Learning and Data Augmentation for Clinical Semantic Textual Similarity Calculation: Algorithm Validation Study

**DOI:** 10.2196/23086

**Published:** 2021-01-22

**Authors:** Junyi Li, Xuejie Zhang, Xiaobing Zhou

**Affiliations:** 1 School of Information Science and Engineering Yunnan University Kunming China

**Keywords:** data augmentation, semisupervised, self-ensemble, ALBERT, clinical semantic textual similarity, algorithm, semantic, model, data sets

## Abstract

**Background:**

In recent years, with increases in the amount of information available and the importance of information screening, increased attention has been paid to the calculation of textual semantic similarity. In the field of medicine, electronic medical records and medical research documents have become important data resources for clinical research. Medical textual semantic similarity calculation has become an urgent problem to be solved.

**Objective:**

This research aims to solve 2 problems—(1) when the size of medical data sets is small, leading to insufficient learning with understanding of the models and (2) when information is lost in the process of long-distance propagation, causing the models to be unable to grasp key information.

**Methods:**

This paper combines a text data augmentation method and a self-ensemble ALBERT model under semisupervised learning to perform clinical textual semantic similarity calculations.

**Results:**

Compared with the methods in the 2019 National Natural Language Processing Clinical Challenges Open Health Natural Language Processing shared task Track on Clinical Semantic Textual Similarity, our method surpasses the best result by 2 percentage points and achieves a Pearson correlation coefficient of 0.92.

**Conclusions:**

When the size of medical data set is small, data augmentation can increase the size of the data set and improved semisupervised learning can boost the learning efficiency of the model. Additionally, self-ensemble methods improve the model performance. Our method had excellent performance and has great potential to improve related medical problems.

## Introduction

With the rapid development of computers and artificial intelligence, information availability has begun to show exponential growth. We are already in an era of information explosion. When faced with a large amount of information, time is wasted screening valid information. In addition, a large amount of information is stored in the form of text. Whether involving cluster storage or referring to related information, efficient information matching and screening is crucial. The importance of text information processing research has become very obvious. With major breakthroughs in the research of related algorithms in natural language processing and artificial intelligence, increasingly, research has been devoted to text information processing.

Textual similarity calculation [1] is a key technology for efficient information screening and matching in the field of text processing. Previous work [2-8] has proposed some methods for textual similarity calculation, for example, traditional text similarity calculation methods [2], word similarity calculation [3], vector space model [4], and latent Dirichlet allocation model [5]. At present, with the development of deep learning and neural networks, methods based on neural networks have become popular, for example, word vector embedding method [6,7] and one-hot representation [8]. At the same time, these methods can also be clinically applied.

In the field of medicine, with the rapid increase in electronic medical data [9], electronic medical records and medical documents have become important data resources for medical clinical research. However, most of these data resources are stored unprocessed or in heterogeneous text formats. To understand the content of text data, it is necessary to integrate structured and heterogeneous clinical data resources, medical records, and scientific research documents. Similarity calculation can improve information retrieval performance for medical resources and effectively allow the integration of heterogeneous clinical data. The concept of semantic similarity evaluation is the key to understanding text data resources, which can effectively allow the processing, classification, and structured processing of those resources. For example, a semantic similarity method can be used to semantically analyze patient medical records to identify similar cases and find the best solution.

**Figure 1:**
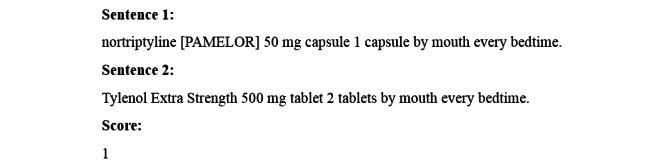
An example from the Clinical STS.

However, a large number of publicly available medical data sets are restricted because of privacy, and there are insufficient sources of medical data sets. The scarcity of data sets has led to the slow development of natural language processing (NLP) in the medical field. In recent years, more researchers have begun to pay attention to this issue. Therefore, competitions related to textual semantic similarity calculation have been produced, such as SemEval [10], to develop an automated method, and the 2019 National NLP Clinical Challenges (N2C2) Open Health Natural Language Processing (OHNLP) [11,12] shared task Track 1 on Clinical Semantic Textual Similarity (STS) [13], for systems based on semisupervised learning. An example of clinical STS is shown in Figure 1. The score indicates the similarity between the 2 sentences are and fall within an ordinal range, ranging from 0 to 5, where 0 means that the 2 sentences are completely different (ie, their meanings do not overlap) and 5 means that the 2 sentences have complete semantic equivalence.

Teams that participated in the 2019 N2C2 OHNLP Clinical STS challenge demonstrated good results with methods such as multitask learning, XLNet, and ClinicalBERT methods. In the challenge, we used recursive neural networks and variants of these neural networks for experiments, such as long short-term memory neural networks [14], convolutional neural networks [15,16], capsule neural networks [17], and ordered long short-term memory neural networks. In addition, we combined some popular deep learning mechanisms, such as attention [18] and Siamese [19,20] networks. Through comparative experimental research, we obtained a Pearson correlation coefficient of 0.66 [21] in the official submission, which was not a satisfying result. Compared with other teams’ methods, our model had 2 drawbacks. First, because the size of clinical data sets was small, there were not enough data to train the model, which led to insufficient learning and understanding of the model. Second, our model was based on a recurrent neural network. Due to the influence of the forget gate in the recurrent neural network, important information may be lost in the process of long-distance propagation, which prevents the model from extracting key information. As a result, the learning efficiency of the model decreased.

To address the abovementioned problems, this paper proposes a self-ensemble [22] ALBERT [23] model under semisupervised learning [24,25] with easy data augmentation (EDA) [26] to calculate the semantic similarity of clinical text.

## Methods

### Overview

In this section, we introduce 3 highlights of our method. Our method uses data augmentation and semisupervised learning to expand the scale of the data set from different levels. We pretrained ALBERT (based on self-ensemble methods) to strengthen the acquisition of key information and improve the performance of the model, and semisupervised learning and data augmentation methods were used to expand the number of data sets and increase the representation of data sets, which can prevent self-ensemble methods from overfitting.

### Data Augmentation

By using external general domain data sets for semisupervised learning, we indirectly solved the problem of insufficient data. However, for medical data, semisupervised learning does not directly increase the amount of medical data. Therefore, we used an EDA method to directly increase the amount of medical data.

Generally, data augmentation is used in computer vision to flip, zoom, and add noise to a picture. These operations can increase small amounts of data, which can help train a more robust model; however, for text data, data augmentation is mainly used for operations such as replacing, adding, and deleting text. Previous work [27,28] has proposed some methods for data augmentation in NLP. For example, a study [27] translated sentences into French and then into English to generate new data. Other work has used data noising as smoothing [28]. However, these methods are highly time- and resource-consuming thus are not often used in practice.

In this paper, we use the form of EDA [26] shown in Table 1. Due to the irreplaceability of proper nouns in medical data, the selection range of the replacement operation has been optimized to keep proper nouns as much as possible. The size of medical data set increased from 1642 to 16,411 after EDA. We can intuitively see a substantial increase in the amount of medical data. We verified that this method increases the size of data set.

**Table 1:** Sentences generated using EDA.

Operation	Sentence 1	Sentence 2	Sentence 3
None^a^	oxycodone [ROXICODONE] 5 mg tablet 0.5-1 tablets by mouth every 4 hours as needed.	A lady is running her cute dog through an agility course.	A beautiful woman with a young girl pose with bear statues in front of a store.
Synonym replacement	oxycodone [ROXICODONE] 5 mg tablet 0.5-1 tablets by mouth every 4 hours as indeed.	A lady is running her cute dog through an legerity course.	A beautiful woman with a young girl pose with bear figurines in front of a store.
Random insertion	oxycodone [ROXICODONE] 5 mg tablet 0.5-1 tablets by every mouth every 4 hours as needed.	A lady is running her cute dog through an amazing agility course.	A beautiful woman with a young girl pose with lovely bear statues in front of a store.
Random deletion	oxycodone [ROXICODONE] 5 mg tablet 0.5-1 tablets by mouth every 4 hours.	A lady is running her dog through an agility course.	A woman with a young girl pose with bear statues in front of a store.

^a^None indicates that this sentence did not undergo any operation.

### Semisupervised Learning

Because there was not a sufficient amount of medical data, the training of the model was not complete. To solve this problem, we used the semisupervised learning method in transfer learning.

The semisupervised [29] pretraining task in NLP is a form of transfer learning that aims to establish a wide range of semantic understanding to promote the performance improvement of training and testing tasks. It has been proven that semisupervised pretraining in transfer learning is very effective in benchmark NLP tasks, and the application prospects in medical NLP tasks are particularly broad. Nonspecific pretraining tasks are used for general medical domain tasks; however, commonly used and publicly available data sets are not specific to the medical domain and may not be well summarized. Therefore, the transfer of nonspecific pretraining tasks and the promotion of language models to medical domain tasks are very important for future model development.

**Figure 2:**
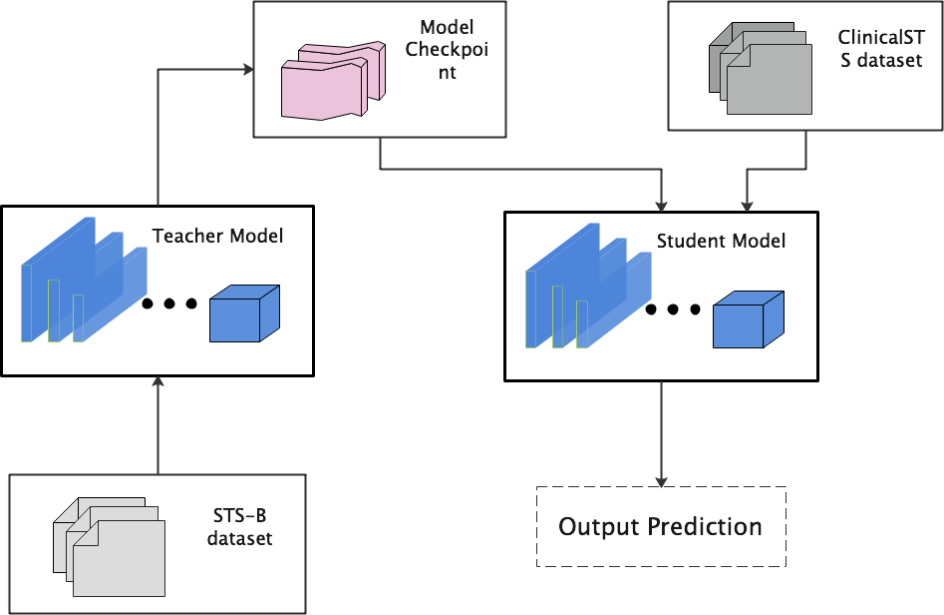
Semisupervised learning.

To improve traditional semisupervised learning, we used the *teacher* and *student* idea in data distillation [30,31] to improve the design of semisupervised learning. Teacher–student refers to the same training process. The beginning of the student's training is the end of the teacher's training, which can deepen the learning of the model. We used the teacher–student approach to design semisupervised learning. The teacher part uses a data set from the common domain, using the STS-B data set from the General Language Understanding Evaluation standard of the general domain. The student part uses a clinical text data set. Our semisupervised learning method is shown in Figure 2.

### Self-Ensemble ALBERT Model

**Figure 3:**
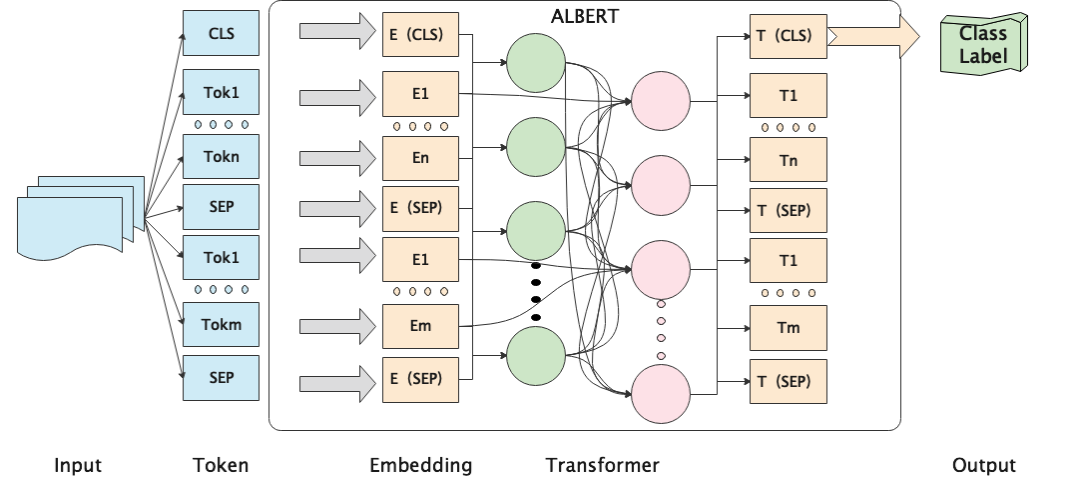
Model architecture.

ALBERT has been applied to some tasks, such as natural language inference [32], sentiment analysis [33], causality analysis [34], and medical machine reading [35]. The self-attention structure is the core part of the transformer mechanism. The self-attention structure can directly calculate the similarity between words, which can intuitively solve the problem of long-distance information dependence. The combined self-attention structure transformer's semantic feature extraction ability is better than those of long short-term memory and convolutional neural networks, and it performs better under the combined action of decomposed embedding parameters and cross-layer shared parameters. Therefore, the pretrained self-attention structure, namely, the pretrained ALBERT model, was applied to our model. ALBERT is a variant of BERT that adds 2 methods of decomposing embedded parameters and sharing parameters across layers. It has 3 improvements. First, ALBERT decomposes embedding, which makes a large number of parameters sparse and reduces the number of dictionaries. Second, ALBERT adopts cross-layer parameter sharing, which reduces the parameter scale and improves the training speed. Third, ALBERT uses intersentence coherence, which makes the model unaffected by specific tasks. The architecture of the ALBERT model is shown in Figure 3.

Following ALBERT, we first embedded the input data. Our embedding representation is constructed by the sum of token embedding, segment embedding, and location embedding. The input sequence is *S* = [*s*_1_, *s*_2_, ..., *s*_n_], where *n* is the number of words in the input. The tokens “[CLS]” and “[SEP]” were added at the beginning and end of each instance, respectively.

Then, we input the data into the ALBERT model, which is made up of *n* transformer stacks,


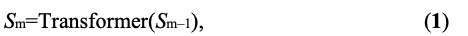


where *S*_m_ is the output of transformer stack *m*.

Since the results do not need to be normalized, we did not use an activation function.

**Figure 4:**
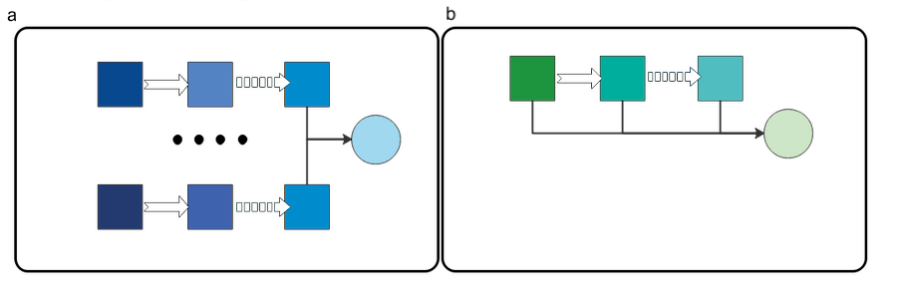
(a) Traditional ensemble vs (b) self-ensemble architecture.

To achieve the best performance, the ALBERT model was fine-tuned. ALBERT models are usually fine-tuned using stochastic gradient descent methods. In fact, fine-tuning the performance of ALBERT is usually sensitive to different random seeds and orders of the training data, especially if the last training sample is noisy. To alleviate this situation, an ensemble method was used to combine multiple fine-tuning models because it can reduce overfitting and improve model generalization. The ensemble ALBERT model usually has better performance than a single ALBERT model. However, training multiple ALBERT models simultaneously is time-consuming. It is often impossible to train multiple models with limited time and GPU resources. Therefore, we improved the model ensemble method to fine-tune the ALBERT model. Our model’s ensemble method is called self-ensemble. The self-ensemble architecture is shown in Figure 4. The formula for self-ensemble is


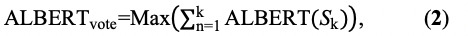


where ALBERT(*S*_k_) represents the checkpoints of the model with *k* training steps.

### Data Sets

The Clinical STS shared task data set was collected from electronic health record in the Mayo Clinic clinical data warehouse. Since the Mayo Clinic has completed the system-wide electronic health record conversion of all care locations from General Electric to Epic, the Clinical STS shared task data set will be extracted from the historical General Electric and Epic systems.

STS-B is a carefully selected English data set used in shared tasks between SemEval and SEM STS between 2012 and 2017. The data was divided into a training set, a development set, and a test set. The development set can be used to design new models and adjust hyperparameters. STS-B can be used to make comparable assessments in different research work and improve the tracking of the latest technology.

Table 2 shows the size of data set in the Clinical STS data set and the STS-B data set. The STS-B data set was used for the semisupervised learning training model. The STS-B data set comes from a data set collected by the general domain criterion General Language Understanding Evaluation. The Clinical STS data set was used to test the experimental results. The Clinical STS data set was provided by the competition organizer.

The STS-B data set provides paired text summaries, which are mainly from STS tasks in SemEval obtained over the years. The Clinical STS data set provides pairs of clinical text summaries, which are sentences extracted from clinical notes. This task assigns a numerical score to each pair of sentences to indicate their semantic similarity. Table 3 shows that the scores fall within an ordinal range, ranging from 0 to 5, where 0 means that the pair of sentences are completely different (ie, their meanings do not overlap) and 5 means that the pair of sentences have complete semantic equivalence.

**Table 2:** The size of data set.

Data set	Training	Validation	Test
STS-B	5749	1500	1379
Clinical STS	1642	N/A^a^	412

**Table 3:** Similarity scores with examples.

Score	Sentence 1	Sentence 2
0	The patient has missed 0 hours of work in the past seven days for issues not related to depression.	In the past year, the patient has the following number of visits: none in the hospital none in the er and one as an outpatient.
1	nortriptyline [PAMELOR] 50 mg capsule 1 capsule by mouth every bedtime.	Tylenol Extra Strength 500 mg tablet 2 tablets by mouth every bedtime.
2	bupropion [WELLBUTRIN XL] 300 mg tablet sustained release 24 hour 1 tablet by mouth one time daily.	Flintstones Complete chewable tablet 1 tablet by mouth two times a day.
3	Given current medication regimen, the following parameters should be monitored by outpatient providers: None	Given current medication regimen, the following parameters should be monitored by outpatient providers: lithium level
4	The diagnosis and treatment plan were explained to the family/caregiver who expressed understanding of the information presented.	Explained diagnosis and treatment plan; patient expressed adequate understanding of the information presented today.
5	Learns best by: verbal instructions as procedure is being performed, reading, seeing, listening.	Learns best by: verbal instruction while procedure is performed, reading, seeing, listening.

### Metric

We used the Pearson correlation coefficient as an evaluation criterion for the performance of the task. The Pearson correlation coefficient,


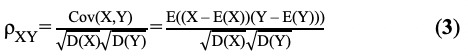


where *E* is the mathematical expectation (or mean), *D* is the variance, and Cov(*X*,*Y*)=E{ [X – E(X)] [Y – E(Y)]} is the covariance of random variables *X* and *Y*, is used to measure the degree of correlation between 2 variables.

### Experimental Setting

In the experiments, we used Intel Xeon 2.2 GHz and Nvidia Tesla V100 32 GHz processors. Since we use semisupervised learning and self-ensemble techniques, our model will be stored by the checkpoint. The input dimensions of each of our data sets are the same. The optimal setting for the length of the input sequence is 64, and the optimal setting for the batch size was 32. The optimal setting for the checkpoint was 200. The optimal setting of the training step was 3598. In the experiments, we did not cross-train on the data set.

## Results

### Performance Comparison

Table 4 shows the top 5 performance results for the 2019 N2C2 OHNLP Track 1 Clinical STS, the value that we obtained during the challenge, and the value obtained by the method presented in this paper. Our current method achieves a good result—the Pearson correlation coefficient value exceeded the best result by 2 percentage points.

**Table 4:** Results on the test set for Clinical STS.

Methods	Pearson correlation coefficient
Multitask learning, ClinicalBERT	0.90
Multitask learning, BERT	0.89
BERT, XLNet	0.88
BERT	0.87
BERT, XLNet	0.87
Our previous method^a^	0.66
Our method in this paper	0.92

^a^Ordered short long-term memory and attention.

### Data Augmentation

The EDA method uses text replacement and deletion operations, optimizes the selection range of replacement and deletion, and retains the medical proper nouns in the data set. Table 5 shows the effect of using EDA on the model performance. After EDA, the size of medical data set is expanded, and the model's performance was greatly improved.

**Table 5:** Comparison between the model with and without EDA.

Methods	Pearson correlation coefficient
Without EDA^a^	0.88
With EDA	0.92

^a^EDA: easy data augmentation.

### Semisupervised Learning

The semisupervised learning method uses the general domain data set STS-B for training to solve the problem of insufficient medical data. Table 6 shows the effect of using semisupervised learning on the model performance. We can see that semisupervised learning can greatly improve the efficiency of the model.

**Table 6:** Comparison between the model with and without semisupervised learning.

Methods	Pearson correlation coefficient
Without semisupervised learning	0.87
With semisupervised learning	0.92

### Self-Ensemble ALBERT

Table 7 shows the effect of using the self-ensemble method on the model performance. We can see that the efficiency of the model with self-ensemble is better than that of the ordinary ensemble model. Additionally, self-ensemble greatly shortens the training time of the model, reduces the calculation time of the algorithm, and improves the efficiency of the algorithm.

BERT and ALBERT are pretrained models with the same self-attention structure. As shown in Table 8, the performance of ALBERT is better than that of BERT on the Clinical STS data set.

**Table 7:** Comparison among the model without ensemble, the model with ensemble, and the model with self-ensemble.

Method	Pearson correlation coefficient
None	0.85
Ensemble^a^	0.89
Self-ensemble	0.92

^a^Ensemble represents an ensemble method through multiple ALBERT models.

**Table 8:** Comparison between the ALBERT and BERT models.

Methods	Runtime (minutes)	Convergence speed^a^ (steps)	Pearson correlation coefficient
BERT	50	3300	0.86
ALBERT	32	2700	0.92

^a^Convergence speed is measured using the training steps.

## Discussion

### Overview

This paper makes the following contributions. First, we used the EDA text data augmentation method. This method increased the number of data through a series of operations and enriched the semantics of the data. Second, for the problem of insufficient medical data, we used a semisupervised learning method. This method relied on the use of external data to enrich the semantics. Third, to solve the problem of learning complex semantics and the loss of key semantic information, we used the self-ensemble ALBERT model for semantic similarity calculation of clinical text. This method not only improves the results of the semantic similarity calculation of clinical text but also, due to the improvement of the self-ensemble of our model, allows the algorithm to shorten its running time and improve its efficiency. With these techniques, our model obtained a Pearson correlation coefficient of 0.92.

In order to test the influence of the method on performance, we conducted ablation experiments on EDA, semisupervised learning, and self-ensemble. At the same time, in order to verify the performance of the model, we also performed ablation experiments on ALBERT.

### Conclusions

Compared with other models and methods, combining an EDA and self-ensemble ALBERT model under semisupervised learning to perform clinical textual semantic similarity calculations can save a large amount of training time and allows more data to be trained at the same time. This brings great convenience for practical applications and scientific research.

In the future, we will study how to combine reinforcement learning to process natural language to further improve the performance of the model and handle the dilemma of bloated or erroneous in electronic health records caused by the increasing use of copy and paste.

## Abbreviations

EDA: easy data augmentation
GLUE: General Language Understanding Evaluation
OHNLP: Open Health Natural Language Processing
N2C2: National NLP Clinical Challenges
NLP: natural language processing
STS: semantic textual similarity
